# Exploring protein–protein ligation approaches for the cytosolic delivery of antigens using AIP56

**DOI:** 10.3389/fcimb.2025.1596550

**Published:** 2025-08-06

**Authors:** Bruno Pinheiro, Ana C. Moura, Pedro Oliveira, Jorge E. Azevedo, Ana do Vale, Nuno M. S. dos Santos

**Affiliations:** ^1^ Fish Immunology and Vaccinology, Instituto de Investigação e Inovação em Saúde (i3S), Universidade do Porto, Porto, Portugal; ^2^ Fish Immunology and Vaccinology, Instituto de Biologia Molecular e Celular (IBMC), Universidade do Porto, Porto, Portugal; ^3^ Doctoral Program in Molecular and Cell Biology (MCBiology), Instituto de Ciências Biomédicas Abel Salazar (ICBAS), Universidade do Porto, Porto, Portugal; ^4^ Instituto de Ciências Biomédicas Abel Salazar (ICBAS), Universidade do Porto, Porto, Portugal; ^5^ Organelle Biogenesis and Function, Instituto de Investigação e Inovação em Saúde (i3S), Universidade do Porto, Porto, Portugal; ^6^ Organelle Biogenesis and Function, Instituto de Biologia Molecular e Celular (IBMC), Universidade do Porto, Porto, Portugal

**Keywords:** AB toxins, biologics, cytosolic delivery, protein-protein fusion, AIP56

## Abstract

**Introduction:**

The intracellular delivery of biologics, particularly large cargoes like proteins, remains a challenge in biotechnology and biomedicine. The modular structure of well-characterized AB toxins allows different cargoes to be grafted, creating a target-specific biotechnological tool capable of cytosolic delivery.

**Methods:**

In this study, we employed protein–protein fusion strategies—SpyCatcher003, SnoopCatcher, and SnoopLigase—to generate chimeras between the delivery region of AIP56 (AIP56^L258-N497^) and β-lactamase and performed functional delivery assays.

**Results:**

The chimeras were successfully obtained using these strategies and were all able to deliver β-lactamase into the cytosol of J774.A1 macrophages. Cellular fractionation showed that, although most of the β-lactamase remains associated with the endosomal compartment, an active portion is released into the cytosol.

**Conclusion:**

AIP56 delivery region transporting other cargo directly to the cytosol of antigen-presenting cells might be a promising platform for antigen/cargo delivery. This study highlights the potential of protein–protein fusion strategies to create versatile, antigenically distinct toxin-based delivery systems for therapeutic applications.

## 1 Introduction

The use of biopharmaceuticals (biologics) for replacement therapy, vaccines, or as agonists/antagonists has a huge potential in therapeutic and prophylactic strategies. Indeed, the demand for biologics has grown steadily in recent years, with a market size estimated to reach 655.6 billion USD by 2030 (IndustryArc, 2024). There are numerous advantages in using biologics, including their high potency and specificity, their diverse molecular targets, and their lower toxicity and tissue accumulation when compared with traditional small-drug molecules (Fosgerau and Hoffmann, 2015; Basak et al., 2022). However, delivering protein-based biologics into the cytosol of eukaryotic cells remains a major challenge, as they cannot be readily transported across the lipid membrane of the cells due to their usually large size, polarity, and complexity. This limitation hinders the full potential of biologics and constrains their use to extracellular targets only. Current vehicles for delivering drugs to the cell cytosol, such as virus-like particles (VLPs) (Zepeda-Cervantes et al., 2020), cell-penetrating peptides (CPPs) (Gessner et al., 2020; Khan et al., 2021), or nanoparticles (NPs) (Yetisgin et al., 2020), lack selectivity to target specific cells and tissues, have poor endosomal escape capacity, and have limited *in-vivo* efficacy (Beilhartz et al., 2017). Therefore, delivery tools that could efficiently and safely transport functional biological molecules into the cytosol of specific cells are a pressing need (Beilhartz et al., 2017; Piot et al., 2021). In the case of vaccination strategies in which the delivery of antigens to the cytosol of antigen-presenting cells (APCs) is a major goal to activate specific cytotoxic T lymphocyte (CTL) responses and achieve effective vaccination against cancer or intracellular pathogens, adjuvants that promote cross-presentation, cross-priming, and consequent CTL response have been used to overcome this limitation (Lee and Suresh, 2022).

An alternative solution to address this challenge may be provided by bacterial AB toxins (Beilhartz et al., 2017; Piot et al., 2021). These toxins are remarkably specific and potent virulence factors secreted by bacteria that act on molecular targets inside eukaryotic cells (Falnes and Sandvig, 2000). They generally display a modular structure comprising two distinct components: component A, containing an enzymatic activity toward molecules with crucial function in eukaryotic cells, and component B, containing a receptor-binding domain or region conferring cell specificity, and a translocation domain or region that assists the translocation of the enzymatic moiety into the cytosol (Falnes and Sandvig, 2000). Components A and B can be encoded in a single gene (i.e., single-chain toxins) or by independent genes whose protein products assemble during or after secretion, yielding multicomponent toxins whose subunits are associated by non-covalent interactions (Geny and Popoff, 2006b, 2006a). After receptor-mediated endocytosis, AB toxins reach their cytosolic target either directly from the endosomal compartment upon acidic pH-triggered unfolding and pore formation (short-trip toxins) or from the endoplasmic reticulum (ER) after retrograde transport from the endosomal compartment to the ER (long-trip toxins). The modular structure confers to AB toxins advantages as biotechnological tools, since different cargoes can be grafted to them to generate chimeric molecules with novel properties (Odumosu et al., 2010; Beilhartz et al., 2017; Piot et al., 2021; Márquez-López and Fanarraga, 2023). Not surprisingly, several AB toxins have already been used to generate useful molecules. For instance, *Pseudomonas* exotoxin A has been engineered as immunotoxins for antitumor therapy, shiga toxin has been engineered for the delivery of cytotoxic drugs in Gb3-positive tumors, and both cholera toxin and *Bordetella pertussis* adenylate cyclase (CyaA) toxin were engineered as antigen delivery platforms to promote highly specific immune responses (Eriksson et al., 2003; Weldon et al., 2009; Batisse et al., 2015; Chenal and Ladant, 2018; Falahatgar et al., 2018; Ladant, 2021).

We have been characterizing AIP56 (Apoptosis Inducing Protein of 56 kDa), a single-chain AB toxin (Do Vale et al., 2005; Silva et al., 2013) that targets sea bass (*Dicentrarchus labrax*) macrophages (Do Vale et al., 2005; Pereira et al., 2014). AIP56 is secreted by the type 2 secretion system of *Photobacterium damselae* subsp. *piscicida* (Do Vale et al., 2017), a Gram-negative bacterium pathogenic for warm-water marine fish that causes high economic losses to the aquaculture sector. The toxin has a three-domain organization (Lisboa et al., 2023): (i) an N-terminal catalytic domain that cleaves NF-kB p65 (Silva et al., 2013), (ii) a small and structurally simple middle domain involved in pore formation, and (iii) a C-terminal domain involved in receptor-binding and pore formation (Silva et al., 2013; Lisboa et al., 2023). After binding to a still unknown host cell receptor, AIP56 is endocytosed (Pereira et al., 2014), and, upon endosomal acidification, protonation of pH-sensing residues located at the carboxyl-terminal portion of the catalytic domain promotes conformational changes that lead to membrane insertion and pore formation (Lisboa et al., 2023), with consequent translocation of the catalytic domain to the cytosol (Pereira et al., 2019). Cleavage of cytosolic NF-kB p65 in intoxicated sea bass macrophages leads to their death by post-apoptotic secondary necrosis (do Vale et al., 2005). Importantly, mouse and human monocytes, macrophages, and dendritic cells (DCs) also internalize AIP56 and undergo p65 cleavage (Pereira et al., 2014; Freitas et al., 2025). Given the tropism of AIP56 for mammalian APCs, which are clinically relevant cell targets for immunization, this toxin may potentially be used as a platform to deliver antigens into the cytosol of APCs and, in this way, serve as a universal vaccination vector.

We have shown previously that AIP56 is able to transport and efficiently translocate genetically fused β-lactamase (Bla) into the cytosol of mouse macrophages (Rodrigues et al., 2019). However, not all antigens can be genetically coupled to toxin-based delivery platforms, because such fusions are often poorly expressed or unstable/insoluble (Beilhartz et al., 2017; Chenal and Ladant, 2018; Piot et al., 2021). In principle, this limitation can be overcome by using “molecular glues.” These ligation systems allow for the separate production of vectors and cargos in their native forms, and their subsequent fusion, enabling the incorporation of synthetic antigens or antigens containing lipidic or glycosidic determinants (Rabideau and Pentelute, 2016; Miyashita et al., 2021; Park et al., 2024).

Systems such as SpyCatcher are based on the Ig-like domains of adhesins from *Streptococcus pyogenes* that contain an internal isopeptide bond between a lysine and an aspartate, the synthesis of which is catalyzed by an opposing glutamate (**Supplementary Figure S1A**) (Zakeri et al., 2012; Li et al., 2014). The Howarth Lab split these domains into a short peptide (SpyTag) containing the reactive aspartate (SpyTag) and a small protein partner (SpyCatcher) containing the reactive lysine and the catalyst glutamate. Both parts can be genetically added to proteins that, when mixed together, spontaneously form an irreversible isopeptide bond very rapidly, thus covalently linking the two moieties (Zakeri et al., 2012). A very similar alternative to SpyCatcher has been developed by the same group from a *Streptococcus pneumoniae* adhesin named SnoopCatcher. In this version, the tag (SnoopTag) contains a reactive lysine, while its protein partner contains a reactive asparagine and the catalyst glutamate (**Supplementary Figure S1B**) (Izoré et al., 2010; Veggiani et al., 2016). By splitting the SnoopCatcher system into a trio so that the three residues involved in the reaction are located in each unit, they also created the SnoopLigase strategy. An enzyme-like protein that contains the catalyst glutamate (SnoopLigase) promotes the ligation of SnoopTagJr (an improved version of SnoopTag) to a small peptide containing the reactive asparagine (DogTag) (**Supplementary Figure S1B**) (Buldun et al., 2018).

In this work, we explored the SpyCatcher003 (improved version of SpyCatcher) (Zakeri et al., 2012; Li et al., 2014; Keeble et al., 2017, 2019), SnoopCatcher (Veggiani et al., 2016), and SnoopLigase (Buldun et al., 2018; Keeble et al., 2023) ligation systems to fuse β-lactamase to the delivery region of AIP56 (AIP56^L258-N497^) and tested the ability of the chimeras to deliver β-lactamase into the cytosol of macrophages as a proof-of-concept for an AIP56-based antigen delivery platform.

## 2 Materials and methods

### 2.1 Cloning

Plasmids used and generated in this work are listed in **Table 1**. β-lactamase plasmids were constructed by amplifying the nucleotide sequence encoding Bla^L19-W286^ from plasmid p327 (**Table 1**) and cloning it into pET28a NcoI/XhoI restriction sites, in frame with a DNA sequence encoding a flexible glycine-glycine-serine-glycine (GGSG) linker and SnoopTagJr (Bla-SnoopT) or SpyTag003 (Bla-SpyT), both introduced by PCR, and a C-terminal 6×His-tag. The construct for the genetic chimera Bla::AIP56 was generated in a previous work (Rodrigues et al., 2019). The construct encoding DogT-AIP56 was obtained by amplifying the nucleotide sequence encoding AIP56^L258-N497^ from pET28AIP56H+ (do Vale et al., 2005) and cloning it into the NcoI/XhoI restriction sites of pET28a, in frame with DogTag at the N-terminus (introduced by PCR) and a C-terminal 6×His-tag. To generate constructs encoding SnoopC-AIP56 and SpyC-AIP56, the AIP56^L258-N497^ nucleotide sequence was amplified from pET28AIP56H+ (do Vale et al., 2005) and cloned into the SacI/XhoI restriction sites of pET28a, in frame with a C-terminal 6×His-tag, yielding pET28a_AIP56^L258-N497^. SnoopCatcher was amplified from pET28a–SnoopCatcher, whereas SpyCatcher003 was amplified from pDEST14-SpyCatcher003. Finally, both sequences were cloned into pET28a_AIP56^L258-N497^ NcoI/SacI restriction sites, yielding pET28a_SnoopC-AIP56 and pET28a_SpyC-AIP56, respectively.

**Table 1:** Plasmids used in this work.

Plasmid	Purpose	Source/Reference
p327	Template to amplify the coding sequence of Bla^L19-W286^	A gift from Dr. Panagiotis Papatheodorou, Ulm University
pET28a_Bla-SnoopT	Expression of Bla^L19-W286^ in frame with SnoopTagJr and 6x HisTag in the C-terminal	This work
pET28a_Bla-SpyT	Expression of Bla^L19-W286^ in frame with SpyTag003 and 6x HisTag in the C-terminal	This work
pET28a_Bla::AIP56	Expression of Bla^L19-W286^ fused to AIP56^L258-N497^ in frame with a 6x HisTag in the C-terminal	Rodrigues et al, 2019
pET28a–SnoopCatcher	Template to amplify the coding sequence of SnoopCatcher	Addgene plasmid #72322; Veggiani et al, 2016
pDEST14-SpyCatcher003	Template to amplify the coding sequence of SpyCatcher003	Addgene plasmid # 133447; Keeble et al, 2019
pET28AIP56H+	Template to amplify the coding sequence of AIP56^L258-N497^	do Vale et al, 2005
pET28a_AIP56^L258-N497^	Backbone to insert SnoopCatcher/SpyCatcher003 coding sequence in frame with AIP56^L258-N497^	This work
pET28a_SnoopC-AIP56	Expression of SnoopCatcher fused to AIP56^L258-N497^ in frame with a 6× HisTag in the C-terminal	This work
pET28a_ SpyC-AIP56	Expression of SpyCatcher003 fused to AIP56^L258-N497^ in frame with a 6× HisTag in the C-terminal	This work
pET28a_DogT-AIP56	Expression of DogTag fused to AIP56^L258-N497^ in frame with a 6× HisTag in the C-terminal	This work
pET28a–His6-SnoopLigase	Expression of AviTag fused to SnoopLigase in frame with a 6× HisTag in the N-terminal	Addgene plasmid #105626; Buldun et al, 2018

### 2.2 Recombinant protein production and purification

Bla-SnoopT and SpyC-AIP56 were expressed in *E. coli* BL21 Star (DE3) (Thermo Fisher Scientific, Waltham, MA, USA). Bla-SpyT was expressed in *E. coli* SoluBL21 (DE3) (AMSBIO, Milton, UK). Bla::AIP56, SnoopC-AIP56, and DogT-AIP56 were expressed in *E. coli* Rosetta (DE3) (Merk). SnoopLigase was expressed in *E. coli* BL21-Codon Plus (DE3) (Agilent Technologies, Santa Clara, CA, USA).

Competent *E. coli* cells were transformed and cultured at 37°C in 1 L of Luria Bertani (LB) broth with shaking (200 rpm). Protein expression was induced at OD600 ~0.6 by adding 0.5 mM isopropyl β-D-1-thiogalactopyranoside (IPTG) and carried out at 17°C for 20h, except for Bla::AIP56 and SnoopLigase, which were expressed for 4h at 17°C and 37°C, respectively. For protein purification, bacterial cells were harvested by centrifugation (3200 g, 30 min, 4°C) and resuspended in 40 ml of 50 mM Tris-HCl pH 8.0, 300 mM NaCl, or 20 mM Tris-HCl pH 8.0, 200 mM NaCl, 5% (v/v) glycerol in the case of Bla^L19-W286^::AIP56^L258-N497^ (Rodrigues et al., 2019). Bacteria were lysed by sonication and centrifuged (35000 g, 30 min, 4°C). Recombinant proteins were purified from the supernatant using nickel-agarose beads by gravity-flow chromatography, followed by size-exclusion chromatography (Superose 12 10/300 GL). Recombinant protein integrity and purity were analyzed by sodium dodecyl-sulfate polyacrylamide gel electrophoresis (SDS-PAGE) and Coomassie blue R-250 staining.

### 2.3 Determination of recombinant protein concentration

The concentration of recombinant proteins was assessed by measuring absorbance at 280 nm using a NanoDrop 1000 and/or NanoDrop One (Thermo Fisher Scientific, Waltham, MA, USA), considering the extinction coefficient and the molecular weight determined using the ProtParam tool available at https://web.expasy.org/protparam/.

### 2.4 *In-vitro* fusion reactions and ligation product purification

Since it has been shown that generation of different chimeras using Spy/Snoop tags requires different molar ratios (Kim et al., 2024; Eom et al., 2025; Sun et al., 2025) and temperatures (Wang et al., 2024) for the completion of the reaction, we tested several conditions.

SpyTag003-SpyCatcher003: The reaction between Bla-SpyT and SpyC-AIP56 was tested at 4°C and 25°C using 1:1, 2:1, and 5:1 (1 unit equals 10 µM) molar ratios in PBS (final volume of 100 μl). After mixing the two proteins, aliquots were removed immediately (0h) or after 1, 2, 4, 8, and 24h incubation, and subjected to SDS-PAGE. The best condition (4h incubation, 25°C, molar ratio 5:1) was upscaled to 500 μl, and the generated Bla::Spy::AIP56 chimera was purified by size-exclusion chromatography (Superdex 75 10/300 GL) in 50 mM Tris-HCl pH 8.0, 300 mM NaCl. Fractions from the three main OD280 nm peaks were collected and analyzed by SDS-PAGE. Bla::Spy::AIP56 chimera was recovered in the second peak, with minimal contamination from single reactants.

SnoopTagJr-SnoopCatcher: The reaction between Bla-SnoopT and SnoopCatcher003AIP56^L258-N497^ was tested at 17°C and 25°C and 1:1, 2:1, and 1:2 molar ratios in PBS (final volume of 100 μl). Aliquots of the reaction were removed immediately after mixing the proteins (0h) or after 1, 2, 4, 6, 8, 12, and 24h incubation, and subjected to SDS-PAGE. The best condition (24h incubation, 25°C, molar ratio 2:1) was upscaled to 500 μl, and the generated Bla::Snoop::AIP56 chimera was purified by size-exclusion chromatography in 50 mM Tris-HCl pH 8.0, 300 mM NaCl (Superose 12 10/300 GL). Fractions of the two main OD280 nm peaks were collected and analyzed by SDS-PAGE. Bla::Snoop::AIP56 chimera was recovered in the first peak, with minimal contamination from single reactants.

SnoopTagJr-DogTag-SnoopLigase: The reaction between Bla-SnoopT, DogT-AIP56, and SnoopLigase was tested at 4°C, 17°C, and 25°C and 1:1:1, 1:2:2, 2:2:1, and 2:1:2 molar ratios in 50 mM Tris-Borate pH 8.0, 10% (v/v) glycerol (final volume of 100 μl). Aliquots of the reaction were removed immediately after mixing the proteins (0h) or after 3, 6, 9, 12, 24, 30, 36, and 48h incubation, and subjected to SDS-PAGE. To purify the Bla::Dog::AIP56 chimera non-covalently bound to SnoopLigase, the best condition (36h incubation, 4°C, molar ratio 2:2:1) was upscaled to 500 μl, and the generated Bla::Dog::AIP56 + SnoopLigase was purified by size-exclusion chromatography (Superose 12 10/300 GL, GE Healthcare, Chicago, IL, USA) in 50 mM Tris pH 8.0, 300 mM NaCl and then dialyzed to 50 mM Tris-Borate pH 8.0, 10% (v/v) glycerol. Two main OD280nm peaks were observed in the chromatography. The corresponding fractions were collected and analyzed by SDS-PAGE. Bla::Dog::AIP56, non-covalently bound to SnoopLigase, was recovered in the first peak, with minimal contamination from single reactants.

Densitometric analysis of product formation at each time point was performed using Fiji Software (Image J version 1.54f). Briefly, pixel intensity profiles for each time point were plotted, and the value obtained at time point 0h was subtracted to eliminate background effects and/or contaminants.

### 2.5 Nitrocefin *in-vitro* cleavage assay

The β-lactamase activities of the proteins were assessed *in vitro* by measuring the linear rate of change in absorbance at 480 nm of nitrocefin. For this, 5 mg of nitrocefin (484400, Merck, Darmstadt, Germany) were dissolved in 500 μl of DMSO to obtain an 8 mM stock solution. The working solution (0.4 mM), obtained by diluting the stock solution in 100 mM sodium phosphate buffer pH 7.4, was aliquoted and stored at −20°C, protected from light. For the assay, 20 µM of nitrocefin was mixed with 5 nM of each protein in a 1 ml plastic cuvette in 100 mM sodium phosphate buffer at pH 7.4. The absorbance of the final solution was measured immediately in a Shimadzu UV 2401PC spectrophotometer at 480 nm every 8 s for 30 min. Curves were plotted using GraphPad Prism 8, and the initial rates were defined as the slope from a linear regression of the first 30 s of reaction.

### 2.6 Sodium dodecyl-sulfate polyacrylamide gel electrophoresis and western blotting

SDS-PAGE was performed using the Laemmli discontinuous buffer system (Laemmli, 1970) in 14% polyacrylamide gels for Coomassie staining or 10% for Western blotting. Samples were subjected to heating for 5 min at 95°C in SDS-PAGE sample buffer (50 mM Tris-HCl pH 8.8, 2% (w/v) SDS, 0.05% (w/v) bromophenol blue, 10% (v/v) glycerol, 2 mM EDTA, and 100 mM DTT) prior to loading.

For Western blotting, cellular fractions were transferred onto nitrocellulose membranes, and transfer efficiency was confirmed by Ponceau S staining. Membranes were blocked for 30 min at room temperature with 5% skimmed milk in T-TBS, followed by incubation for 1h at room temperature with the primary antibodies against glyceraldehyde-3-phosphate dehydrogenase (GAPDH; clone 6C5, sc-32233) or transferrin receptor 1 (TFR1; clone H68.4, ProteinTech 65236, Rosemont, IL, USA), both diluted in blocking buffer. GAPDH was used as a cytosolic marker, while TFR1 was used as a marker of the plasma membrane fraction. The membranes were washed and incubated for 1h at room temperature with anti-mouse alkaline phosphatase-conjugated secondary antibody diluted in blocking buffer. Immunoreactive bands were detected using NBT/BCIP (Promega, Madison, WI, USA).

### 2.7 Fluorescence resonance energy transfer–based assay

J774A.1 cells, obtained from the American Type Culture Collection (ATCC TIB-67, Manassas, VA, USA), were cultured at 37°C in a humidified chamber and 5% CO2 atmosphere in Dulbecco’s modified Eagle’s medium (DMEM) containing 10% (v/v) inactivated fetal bovine serum (FBS). Cells were seeded at a density of 1 × 10^4^ cells per well in ibidi, Gräfelfing, Germany µ-Slide 8-well plates in DMEM containing 10% (v/v) FBS and allowed to attach and grow for 48h until reaching 60%–70% confluency. The cells were then washed twice with DMEM and incubated with 1 µM of CCF4-AM in Hanks’ balanced salt solution (HBSS) for 30 min at room temperature. After washing twice with HBSS, the β-lactamase-containing proteins were added at a final concentration of 25 nM. The cells were kept on ice for 15 min and then incubated at 37°C for 45 min. Finally, cells were washed twice with Dulbecco’s phosphate buffered saline (DPBS) and fixed on ice for 15 min in 4% (wt/vol) paraformaldehyde in DPBS.

Fixed cells were observed with a CFI PL APO LAMBDA 40X/0.95 objective in a Nikon Eclipse Ti-E microscope. The samples were illuminated by a 395 nm LED by a SpectraX light engine using a quad dichroic filter 310DA/FI/TR/CY5-A and emission filters 450/50 and 525/50. Images were acquired with an EMCCD camera, iXon ULTRA 888. Three independent experiments were performed, and in each experiment, a minimum of 10 microscopic fields were analyzed per condition.

Quantification of cells positive for cleaved CCF4-AM was made using custom-made ImageJ macros on Fiji software (https://osf.io/2nc5j) (Schindelin et al., 2012).

The proportions of positive cells after treatment with the chimeras were compared to the proportions of positive cells after treatment with their β-lactamase counterparts using the chi-square test in IBM SPSS Statistics software. Differences were considered significant when *p* ≤ 0.05.

### 2.8 β-lactamase subcellular release assessment

J774A.1 cells were cultured as described above. Cells were seeded in a T25 flask in DMEM containing 10% (v/v) FBS and allowed to attach and grow for 48h until reaching at least 80% confluency. The cells were then washed twice with PBS and incubated with 25 nM of Bla::Snoop::AIP56 or vehicle for 15 min on ice and then either kept on ice to block endocytosis (negative control) or transferred to 37°C for 45 min. After washing twice with PBS, cells were incubated for 30 min with 200 μg/ml of Proteinase K on ice, washed twice with PBS, and incubated with 250 μg/ml phenylmethylsulfonyl fluoride (PMSF) for 5 min on ice. After washing with PBS, subcellular fractions were obtained based on previously established protocols (Rodrigues et al., 2016; Diaz et al., 1989). Briefly, cells were scraped from the flask in 2 ml of ice-cold SEM buffer (0.25 M sucrose, 1 mM EDTA-NaOH, pH 8.0, and 20 mM MOPS-KOH, pH 7.2) supplemented with 2 μg/ml of E-64 protease inhibitor. Cells were then pelleted by centrifugation (4°C, 5 min, 600 g) and resuspended in four volumes of supplemented SEM buffer. Cells were homogenized on ice, with the help of a G22 Hamilton syringe, and then centrifuged as before. The supernatant (post-nuclear supernatant [PNS]) was diluted two times in buffer P (0.25 M sucrose, 50 mM KCl, 3 mM MgCl_2_, in 20 mM MOPS, pH 7.2) and then centrifuged at 4°C for 30 min at 12,000 g. The supernatant (cytosolic fraction) was collected, while the pellet (organelle fraction) was resuspended in the original volume of buffer P.

To determine the subcellular distribution of β-lactamase, 100 μl of each fraction were mixed with 200 μl of a solution containing 100 mM sodium phosphate buffer pH 7.4, 1% Triton X-100, 2 μg/mL of E-64 protease inhibitor, and 160 μM of nitrocefin and incubated at room temperature in a 96-well plate. After 3h of incubation, β-lactamase activity was assessed by measuring absorbance at 486 nm using a Synergy 2 microplate reader.

## 3 Results

### 3.1 β-lactamase can be efficiently coupled to AIP56 delivery region using biochemical approaches

β-lactamase is an enzyme produced by many bacteria (Kaderabkova et al., 2022), with no known mammalian homologues. The enzyme can be fused to the N terminus or C terminus of most proteins without compromising its activity and can be detected in eukaryotic cells without much background interference (Moore et al., 1997; Zlokarnik et al., 1998; Zlokarnik, 2000; Campbell, 2004). Here, we used three protein–protein ligation systems to fuse β-lactamase to AIP56^L258-N497^ (middle and receptor-binding domains) as an alternative to genetic fusion. Since it has been previously shown that inactivation of the catalytic center renders AIP56 nontoxic (Silva et al., 2013), the absence of the catalytic domain in the chimeras prevents toxicity associated with AIP56.

**Figure 1:**
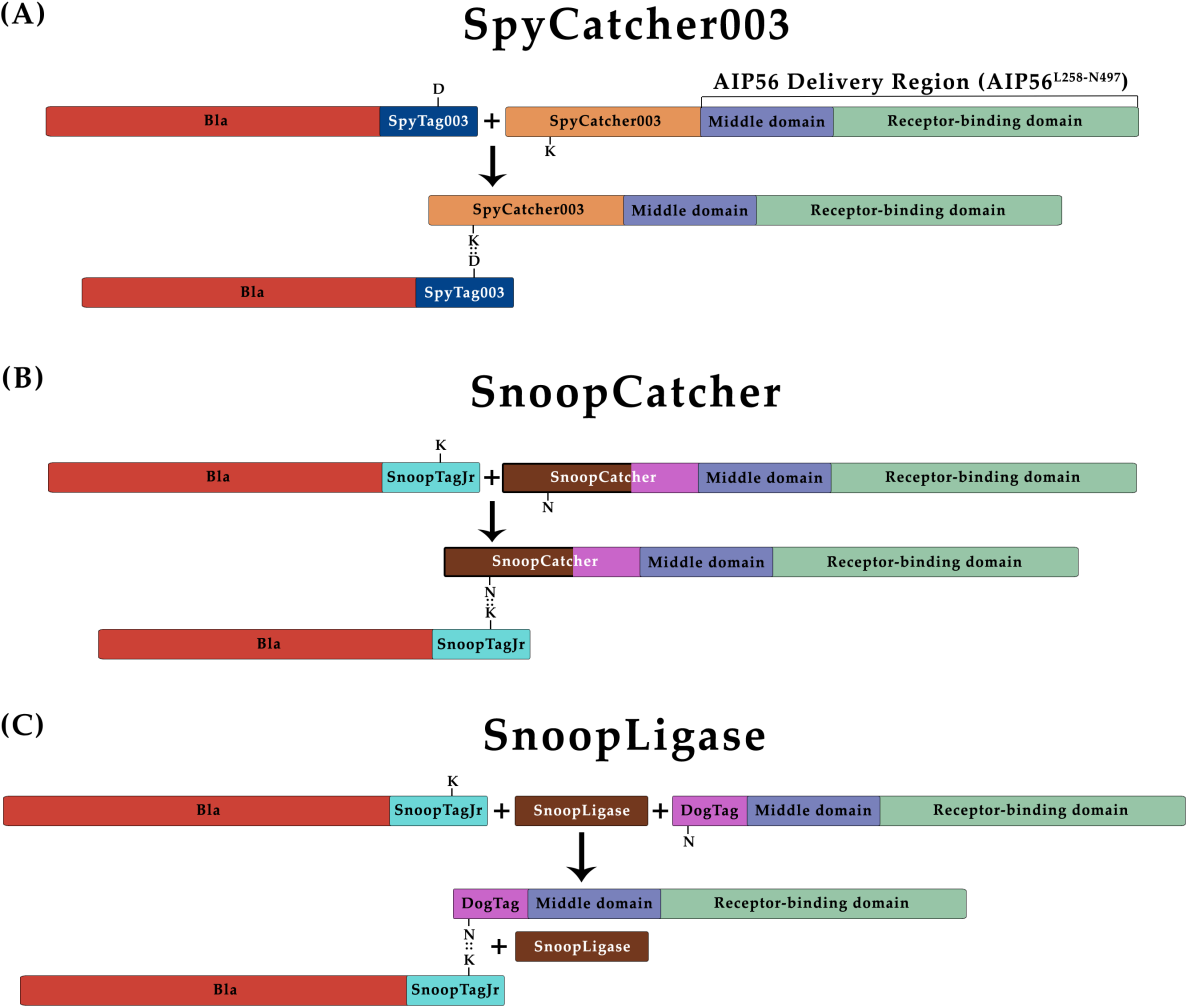
Schematic representation of protein–protein ligation reactions. All proteins were produced with the respective tags/protein partners, and incubated together to create Bla::AIP56^L258-N497^ chimeras. **(A)** SpyCatcher003. D: Aspartate 556; K: Lysine 470. These residues are involved in the formation of the isopeptidic bond between SpyTag003 and SpyCatcher003. **(B)** SnoopCatcher. K: Lysine 742; N: Asparagine 854. These residues are involved in the formation of the isopeptidic bond between SnoopTagJr and SnoopCatcher. **(C)** SnoopLigase. K: Lysine 742; N: Asparagine 854. These residues are involved in the formation of the isopeptidic bond between SnoopTagJr and DogTag.

To obtain Bla^L19-W286^::AIP56^L258-N497^ chimeras through protein–protein fusion approaches, each specific tag and protein partner was genetically fused to Bla^L19-W286^ or AIP56^L258-N497^, respectively. Specifically, (i) for the SpyCatcher003 reaction (**Figure 1A**), SpyTag003 was added to the C terminus of Bla^L19-W286^ (Bla-SpyT), and the SpyCatcher003 was fused to the N terminus of AIP56^L258-N497^ (SpyC-AIP56) (Keeble et al., 2019); (ii) for the SnoopCatcher reaction (**Figure 1B**), SnoopTagJr was added to the C terminus of Bla^L19-W286^ (Bla-SnoopT), whereas SnoopCatcher was fused to the N terminus of AIP56^L258-N497^ (SnoopC-AIP56) (Veggiani et al., 2016); and (iii) for the SnoopLigase reaction (**Figure 1C**), the DogTag was added to the N terminus of AIP56^L258-N497^ (DogT-AIP56) for subsequent reaction with Bla-SnoopT, catalyzed by the SnoopLigase (Buldun et al., 2018). All the proteins were successfully expressed in *Escherichia coli* and purified by Ni-NTA affinity chromatography followed by size-exclusion chromatography (**Supplementary Figure S2**).

**Figure 2:**
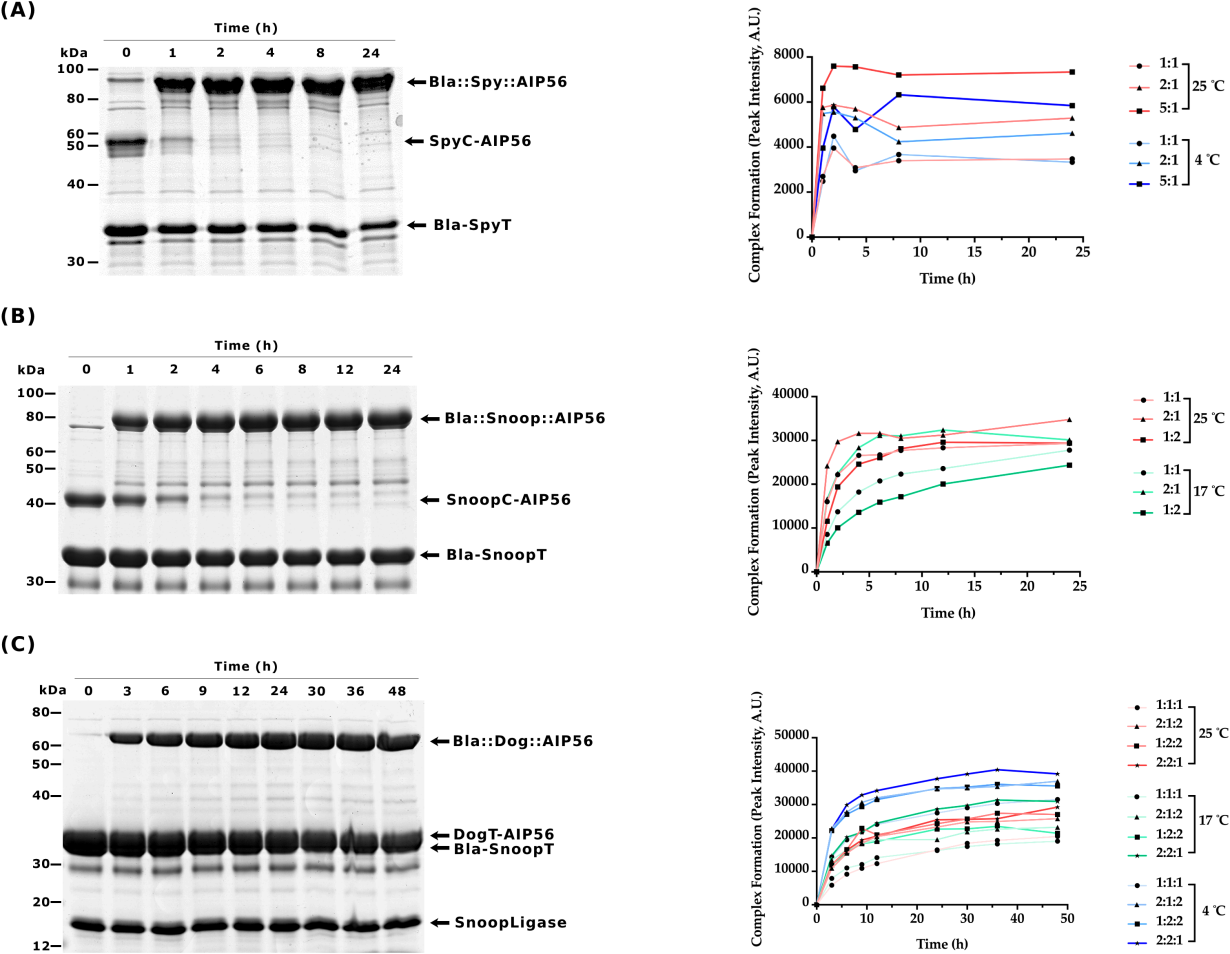
Time-course analyses of ligation reactions between β-lactamase and AIP56^L258-N497^ **(A)** sodium dodecyl-sulfate polyacrylamide gel electrophoresis (SDS-PAGE) of the SpyCatcher003 reaction (left panel) and its quantification (right panel). The gel shows the results obtained with the best condition: 5:1 molar ratio (50 µM:10 µM) of Bla-SpyT to SpyC-AIP56 at 25°C, followed during 24h. Other reaction conditions are shown in **Supplementary Figure S3**. Samples were analyzed by SDS-PAGE followed by Coomassie-blue R-250 staining. Bla-SpyT (32.6 kDa) reacted with SpyC-AIP56 (41.3 kDa) to originate Bla::Spy::AIP56 (73.9 kDa). **(B)** SDS-PAGE of the SnoopCatcher reaction (left panel) and its quantification (right panel). The gel shows the results obtained with the best condition: 2:1 molar ratio (20 µM:10 µM) of Bla-SnoopT to SnoopC-AIP56 at 25°C, followed during 24h. Other experimental conditions are shown in **Supplementary Figure S4**. Bla-SnoopT (31.8 kDa) reacted with SnoopC-AIP56 (41.2 kDa) to originate the Bla::Snoop::AIP56 (73.0 kDa). **(C)** SnoopLigase reaction (left panel) and its quantification (right panel). The gel shows the best condition: 2:2:1 molar ratio (20 µM:20 µM:10 µM) of Bla-SnoopT to DogT-AIP56 to SnoopLigase at 4°C, followed during 48h. Other conditions are shown in **Supplementary Figure S5**. Bla-SnoopT (31.8 kDa) reacted with DogT-AIP56 (31.1 kDa) catalyzed by SnoopLigase (15.4 kDa) to originate the Bla::Dog::AIP56 (62.9 kDa).

For the SpyCatcher-SpyTag reaction, Bla-SpyT was conjugated to SpyC-AIP56 at 25°C or 4°C, using several molar ratios of the proteins. A 5:1 ratio resulted in the highest yield of Bla::Spy::AIP56, with the reaction at 25°C reaching nearly completion after 2h of incubation, as shown by the depletion of SpyC-AIP56 (**Figure 2A** and **Supplementary Figure S3**).

Slightly different experimental conditions were tested for the SnoopCatcher-SnoopTagJr reaction. Specifically, Bla-SnoopT was conjugated to SnoopC-AIP56 at 25°C or 17°C. A 2:1 ratio resulted in a higher yield of Bla::Snoop::AIP56, with the reaction performed at 25°C, reaching nearly completion after 4h of incubation, as shown by the depletion of SnoopC-AIP56 at this time point (**Figure 2B** and **Supplementary Figure S4**).

The experimental conditions used for the DogTag-SnoopTagJr reaction were the following: Bla-SnoopT was mixed with DogT-AIP56 at different molar ratios in the presence of SnoopLigase and incubated at 25°C, 17°C, or 4°C. The best yield was obtained at 4°C using a Bla-SnoopT:DogT-AIP56:SnoopLigase molar ratio of 2:2:1, respectively. Under these conditions, the reaction reached a plateau at the 24h time point. Complete conversion of the reactive proteins into the chimera was not achieved, even after 48h of incubation (**Figure 2C** and **Supplementary Figure S5**).

All the protein chimeras were purified by SEC (**Supplementary Figure S6**) and tested for activity as described below.

### 3.2 Bla::Spy::AIP56, Bla::Snoop::AIP56, and Bla::Dog::AIP56 + SnoopLigase retain β-lactamase activity

**Figure 3:**
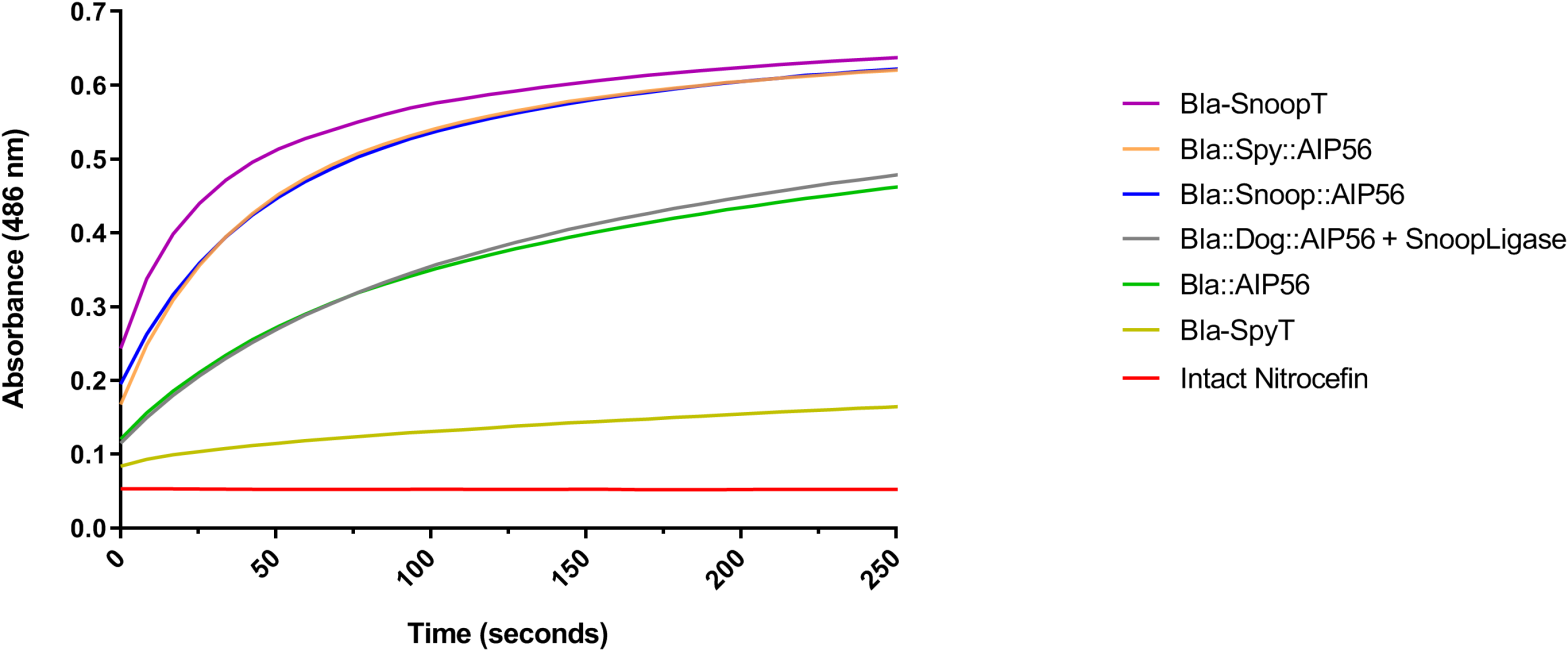
Nitrocefin hydrolysis by Bla^L19-W286^-containing proteins. Each protein (5 nM) was incubated with 20 µM of nitrocefin and absorbance was measured at 486 nm for 250 s.

As a preliminary control to assess whether the tagged β-lactamases (Bla-SpyT, Bla-SnoopT) and the β-lactamase containing chimeras retained enzymatic activity, an *in-vitro* nitrocefin colorimetric cleavage assay was conducted. As a control, the genetic chimera Bla::AIP56 was also included in these assays. Nitrocefin harbors a beta-lactam ring, which is hydrolyzed by beta-lactamase. The hydrolysis reaction can be followed spectrophotometrically by measuring the increase of the absorbance at 486 nm (O’Callaghan et al., 1972). The progress curves obtained reveal that all proteins tested possessed β-lactamase activity (**Figure 3**). To compare activities among proteins, the slopes in the linear part of the graphs (the first 30 s of reaction) were calculated (**Table 2**).

**Table 2:** Relative activities of the Bla^L19-W286^-containing proteins.

Protein	Slope (ΔAbs.min^−1^)
Bla-SpyT	0.77 × 10^−3^
Bla-SnoopT	7.66 × 10^−3^
Bla::Spy::AIP56	7.38 × 10^−3^
Bla::Snoop::AIP56	6.43 × 10^−3^
Bla::Dog::AIP56 + SnoopLigase	3.58 × 10^−3^
Bla::AIP56	3.54 × 10^−3^

### 3.3 All chimeras obtained by protein ligation transport β-lactamase into the cytosol of macrophages

**Figure 4:**
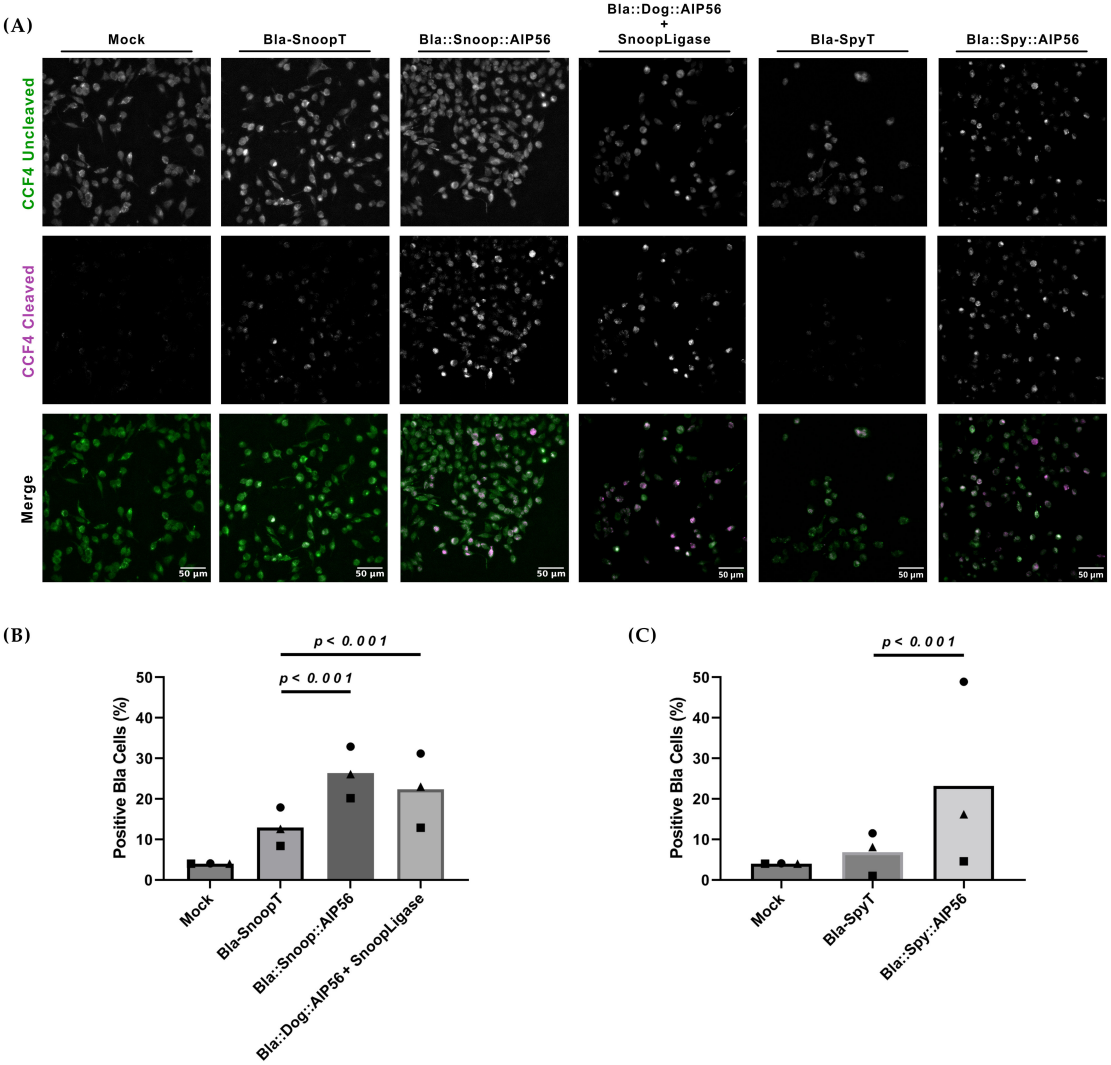
Bla::AIP56^L258-N497^ chimeras obtained by protein ligation deliver β-lactamase into the cytosol of J774.A1 murine macrophages. **(A)** Representative images of macrophages loaded with CCF4-AM and treated with the indicated chimeras. Cells were loaded with 1 µM CCF4-AM before incubation with the indicated proteins (25 nM) for 15 min on ice followed by 45 min at 37°C. Uncleaved and cleaved CCF4-AM signals were detected by fluorescence microscopy shown correspond to representative microscopic fields obtained in one of three independent experiments. **(B)** Quantification of cells positive for cleaved CCF4-AM. Fluorescence intensities corresponding to the cleaved CCF4-AM signal were measured at the single-cell level from three independent experiments. In each experiment, background fluorescence was subtracted from individual cell values. A positivity threshold was then defined based on the vehicle-only control condition. Cells with signal intensities above this threshold were classified as positive. The percentage of positive cells was calculated for each condition. Statistical comparisons were performed using the chi-squared test (see **Supplementary Figure S8**). Differences were considered significant when *p* ≤ 0.05.

Toxin-mediated translocation of β-lactamase across the endosomal membrane can be assessed by a Fluorescence Resonance Energy Transfer (FRET)–based assay (Zuverink and Barbieri, 2015) (**Supplementary Figure S7**). The assay is based on a cell-permeable substrate (CCF4-AM) that consists of a coumarin moiety connected by a β-lactam ring to fluorescein. Excitation of coumarin at 409 nm results in FRET to fluorescein with green fluorescence emission at 520 nm. Upon cleavage of the β-lactam ring by β-lactamase, FRET is disrupted, and excitation of coumarin at 409 nm results in blue fluorescence emission at 447 nm. We have previously used this assay to show that a Bla::AIP56 chimera, obtained by genetic fusion, is able to deliver β-lactamase into the cytosol of mouse bone marrow-derived macrophages (mBMDM) (Rodrigues et al., 2019; Lisboa et al., 2023). Here, we used this assay to determine whether BlaAIP56 chimeras, obtained by protein ligation approaches, are capable of delivering β-lactamase into the cytosol of mouse macrophages. Briefly, J774.A1 macrophages were loaded with CCF4-AM and were then either mock-treated or incubated with Bla::Snoop::AIP56, Bla::Dog::AIP56 + SnoopLigase, Bla::Spy::AIP56, or Bla-SnoopT and Bla-SpyT as negative controls. Cleavage of CCF4-AM was analyzed by fluorescence microscopy, and positive cells were counted to assess delivery. As shown in **Figure 4**, all the chimeras tested led to FRET disruption when compared to cells treated with Bla-SnoopT or Bla-SpyT (*p* < 0.001; **Supplementary Figure S8**).

### 3.4 β-lactamase is released into the cytosol from the Bla::Snoop::AIP56 chimera

**Figure 5:**
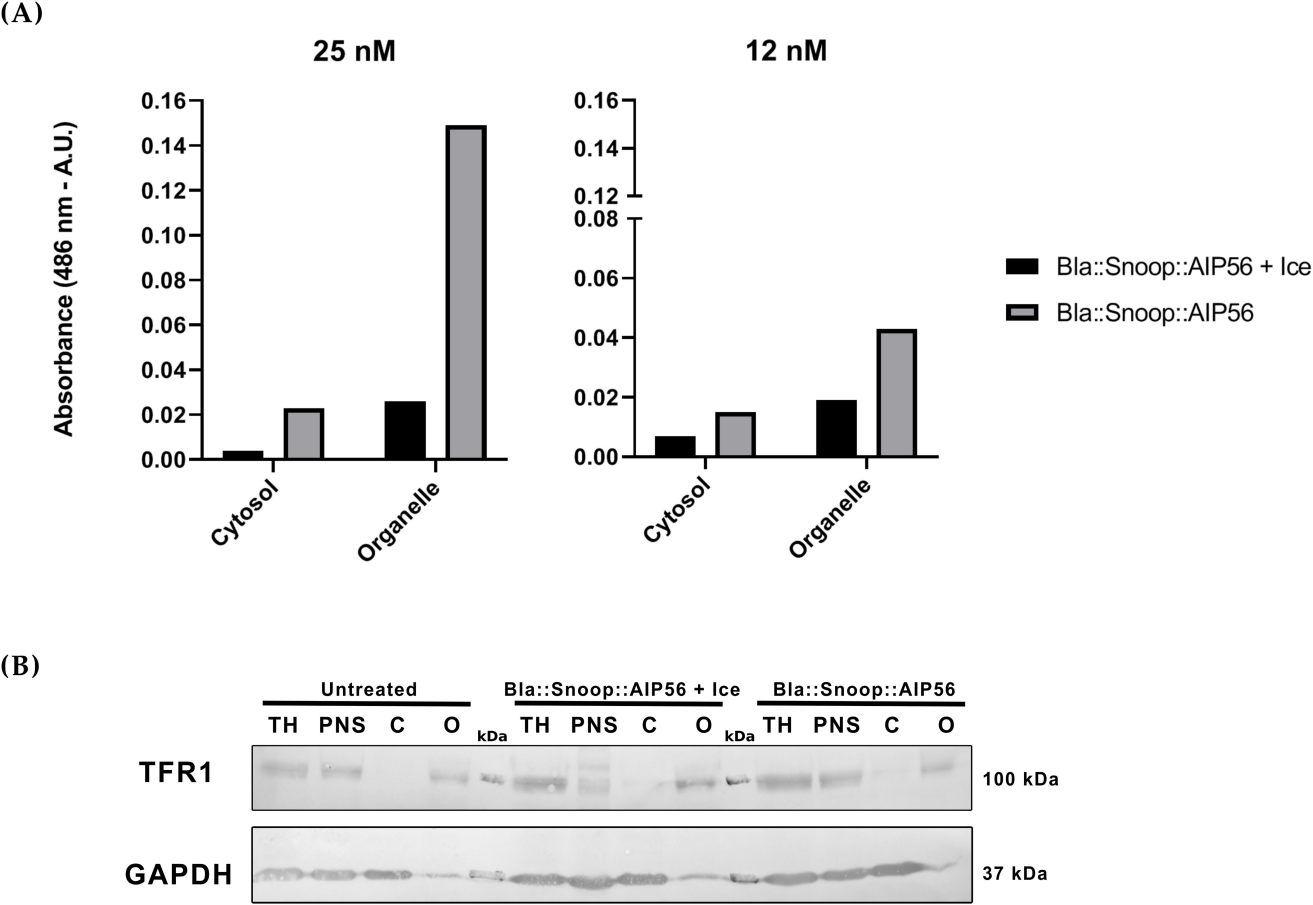
Bla::Snoop::AIP56 releases Bla into the cytosol. **(A)** Nitrocefin cleavage assay. Cells were incubated with 25 nM of Bla::Snoop::AIP56 for 15 min on ice and either maintained on ice (to block endocytosis) or incubated for 45 min at 37°C. or. Cytosolic and organelle fractions were prepared and incubated with 160 μM of nitrocefin to measureβ-lactamase activity. **(B)** Representative Western blot of subcellular fractioning. Subcellular fractions of total homogenate (TH), post-nuclear supernatant (PNS), cytosolic fraction (C), and organelle fraction (O) were subjected to SDS-PAGE and subsequently transferred to a nitrocellulose membrane. Detection of TFR1 and GAPDH (markers for endosomal membrane and cytosol, respectively) was performed using anti-TFR1 and anti-GAPDH antibodies, followed by alkaline phosphatase-based detection with NBT/BCIP. Molecular weight markers (in kDa) were marked in pencil prior to incubation with antibodies.

An important characteristic of an intracellular delivery platform is the ability to release soluble molecules into the cytosol of cells. Although antigen presentation does not strictly require release of the antigen into the cytosol (Zhang et al., 2023), other biologics may need to reach the cytosol in order to access their targets. To determine whether β-lactamase is released into the cytosol, cytosolic and organelle fractions were isolated from cells intoxicated with Bla::Snoop::AIP56 and incubated with nitrocefin to assess the presence of β-lactamase activity. As shown in **Figure 5**, incubation of subcellular fractions from cells intoxicated with 25 nM Bla::Snoop::AIP56 with nitrocefin resulted in increased substrate cleavage compared to the control condition, in which cells were incubated with the chimera on ice to block endocytosis. Similar results were obtained with a dose of 12 nM. Together, these findings indicate that while a substantial portion of β-lactamase remains associated with endosomal membranes or compartments, a small fraction was successfully translocated and reached the cytosol. These results strongly suggest that the delivery region of AIP56 is capable of mediating the cytosolic release of β-lactamase.

## 4 Discussion

The increased use of biologics has been accompanied by the development of various vectors for their delivery, such as VLPs, CPPs, or NPs (Gessner et al., 2020; Yetisgin et al., 2020; Zepeda-Cervantes et al., 2020; Khan et al., 2021). Although these vectors have their own advantages, they all lack one or another feature, such as poorly understood mechanisms of action, low selectivity, fast/slow metabolic clearance, or difficult encapsulation for delivery (McNaughton et al., 2009; Dhindwal et al., 2019; Gessner et al., 2020; Khan et al., 2021; Mitchell et al., 2021; Lakshmanan et al., 2022). Overcoming those problems is challenging but would improve the delivery of biologics without compromising their high efficacy and low toxicity. A system capable of delivering large cargoes, such as proteins, to the cell cytosol would be of immense value in several areas of biotechnology and biomedicine, such as in infection, vaccination, immunomodulation, and anti-tumor activity. In this context, AB toxins rise as valid alternatives for the delivery of biological cargoes. In contrast to other delivery vectors (Gessner et al., 2020; Yetisgin et al., 2020; Zepeda-Cervantes et al., 2020; Khan et al., 2021), the structure and function mechanisms of many AB toxins are relatively well characterized, enabling their engineering to deliver different cargoes into the cytosol of target cells. In fact, several toxins are already being used as delivery tools with some success (Beddoe et al., 2010; Odumosu et al., 2010; Akbari et al., 2017; Kenworthy et al., 2021; Piot et al., 2021), although most of the toxin-cargo chimeras used to date have been produced by fusion at the genetic level. However, this approach is not always possible, due to low expression or solubility problems of the chimeras. An alternative to overcome this problem is to use chemical ligation strategies to connect the delivery moiety of the toxin to the desired cargoes. Protein–protein fusion techniques mediated by sortase A or by SpyCatcher-SpyTag have recently been applied to couple cargoes to toxins or to replace the receptor-binding domain of a toxin by a new ligand that confers a distinct cellular specificity (Park et al., 2020, 2024; Miyashita et al., 2021).

We have previously shown that the middle and receptor-binding domains of AIP56 (AIP56^L258-N497^) are sufficient to translocate β-lactamase from the endosomal compartment to the cytosol of macrophages by using chimeras produced through genetic engineering (Rodrigues et al., 2019; Lisboa et al., 2023). This, together with the fact that AIP56 targets mammalian APCs (Freitas et al., 2025), supports the use of AIP56^L258-N497^ as a platform for delivering antigens to APCs, similarly to what has been shown for CyaA, another AB toxin that also targets APCs (Chenal and Ladant, 2018; Ladant, 2021). However, contrary to CyaA and other AB toxins, which need to be previously activated by cleavage (“nicking”) of a linker loop before triggering the translocation of their cargoes to the cytosol (Gordon and Leppla, 1994; Young and Collier, 2007; Wernick et al., 2010; Uribe et al., 2013), AIP56 is ready to deliver its cargoes without any activation step (Silva et al., 2013; Pereira et al., 2014). Furthermore, in the case of CyA (1706 amino acids long) antigens have been grafted to the catalytic domain of the full-length toxin (Chenal and Ladant, 2018), resulting in chimeras of substantial size, whereas in AIP56, the catalytic domain is replaced by the cargo, resulting in much smaller chimeras, with implications for production and immunogenicity. Moreover, CyaA inserts in the cell membrane, triggering potassium efflux as well as the formation of cation-specific pores that ultimately cause lysis of the APCs (Chenal and Ladant, 2018), posing limits to the dosage and compromising the immune response to the antigen. In contrast, AIP56 translocates only from endosomes (Pereira et al., 2014), and its delivery domains can be used without harm to APCs. However, since the pore-formation mechanism for AIP56 is not completely known, it is difficult to predict how the complexity or size of the cargoes impacts the efficiency of translocation. In fact, although it is known that diphtheria toxin translocates diverse cargoes of different sizes and complexities (Ren et al., 1999; Wu et al., 2006), C2 toxin and anthrax toxin have some limitations transporting stably folded proteins or proteins without an N-terminal positive net charge (Heber et al., 2023). Another relevant aspect to investigate is the stability of the purified chimeras during long-term storage, as this is important for future applications of this technology.

An important condition for the success of a toxin-based vaccination platform is the existence of pre-acquired antigenicity to the toxin-vector or antigenicity acquired during vaccination, which may prevent its use, especially when multiple inoculations are required. Although mutation of immunogenic epitopes or administration of immunosuppressants have been used in protein-based therapies to solve the antigenicity problem (Fosgerau and Hoffmann, 2015; Basak et al., 2022), mutating the right epitopes can be challenging, and immunosuppressants have unwanted side effects and should be avoided when the objective is to induce an immune response against an antigen. Although bacterial toxins may be highly immunogenic by nature (Elson, 1989; Yu et al., 2016; Mazor and Pastan, 2020; Carr et al., 2021), the producing agent of AIP56, *Photobacterium damselae* subsp. *piscicida*, is a well-recognized fish pathogen that does not infect mammals, rendering neutralization of AIP56 by antibodies generated during previous contact with this pathogen unlikely. Even if it were to occur, the use of protein–protein fusion techniques would allow quick coupling of antigens (or drugs) to delivery platforms based on different toxins prepared in advance for this purpose.

In addition to the many AB toxins already studied, there are several uncharacterized toxins in different species of prokaryotes and eukaryotes whose three-dimensional structures predicted by AlphaFold are identical to the crystallographic structure of AIP56 (AIP56-like toxins) or contain a domain similar to the receptor-binding domain of AIP56 (AIP56-related toxins) (Lisboa et al., 2023). Moreover, such proteins present high amino acid similarity, indicating a common evolutionary pathway. Rabbit antiserum against AIP56 does not cross-react with some of these toxins (**Supplementary Figure S9**). Therefore, we hypothesize that the middle and receptor-binding domains of those toxins could also be used as a viable and/or complementary alternative to the AIP56-based platform in cases where neutralizing antibodies are produced against the main vehicle. Of utmost importance to establishing the vaccine application potential of this approach will be to assess whether antigen-AIP56 chimeras will effectively trigger an antigen-specific protective immune response.

In this work, we successfully generated chimeras by fusing the AIP56 delivery region to β-lactamase using SpyCatcher003, SnoopCatcher, and SnoopLigase ligation strategies. More importantly, incubation of mouse J774.A1 with the chimeras led to cytosolic delivery of β-lactamase. Subcellular fractionation of cells treated with the Bla::Snoop::AIP56 chimera confirmed that β-lactamase is released into the cytosol. These findings indicate that AIP56 can be used not only to deliver antigens into antigen-presenting cells (APCs), but also to transport other types of cargo that require free access to the cytosol to reach their molecular targets.

In summary, our data indicate that protein–protein fusion strategies, although more laborious, are feasible and valid alternatives to genetic fusion strategies, particularly when the latter approach is unsuitable. We anticipate that by extending these strategies to other AB toxins, including a range of toxins homologous to AIP56, it will be possible to create a “toolbox” with functionally equivalent but antigenically different interchangeable delivery vehicles ready to couple different antigens.

## Data Availability

The datasets presented in this study can be found in online repositories. The names of the repository/repositories and accession number(s) can be found below: Open Science Framework (https://osf.io/2nc5j) (https://doi.org/10.17605/OSF.IO/2NC5J).
