# Supplementary material for: Exploring protein–protein ligation approaches for the cytosolic delivery of antigens using AIP56
Source: Front Cell Infect Microbiol. 2025 Aug 6;15:1596550. doi: 10.3389/fcimb.2025.1596550 (PMC12364934; doi:10.3389/fcimb.2025.1596550)
Supplement: Supplementary file 1 [file DataSheet1.pdf]

Supplementary Material

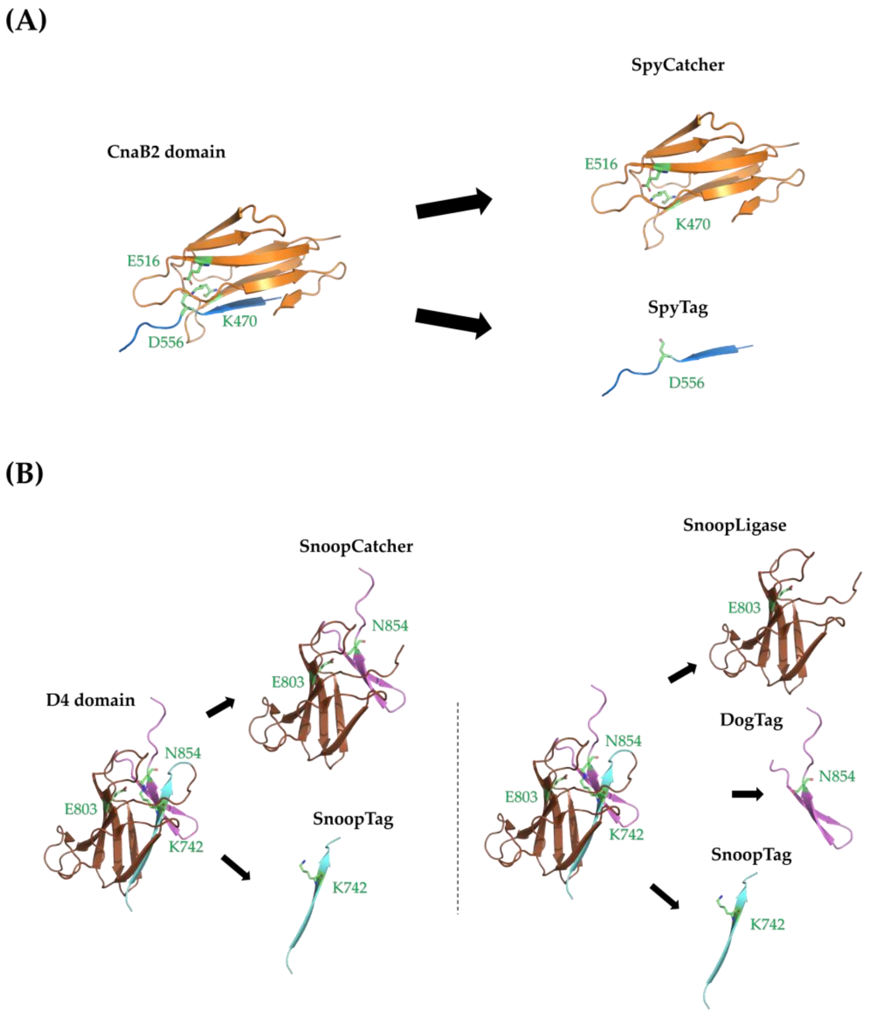

Supplementary Figure 1. Cartoon representation of the SpyCatcher, SnoopCatcher and SnoopLigase fusion systems. (A) The SpyCatcher ligation system. SpyCatcher (orange) and SpyTag (blue) represent two regions of the CnaB2 Ig-like domain from *Streptococcus pyogenes* FbaB adhesin (PDB: 4MLI). The reactive residues in SpyCatcher (K470) and SpyTag (D556) and the catalytic residue in SpyCatcher (E516) are indicated as green sticks. **(B) The SnoopCatcher and SnoopLigase ligation systems.** The Ig-like D4 domain from *Streptococcus pneumoniae* RrgA adhesin (PDB: 2WW8) and the regions corresponding to SnoopCatcher (brown+violet), SnoopTag (cyan), SnoopLigase (brown) and DogTag (violet) are shown. The reactive residues in SnoopCatcher/DogTag (N854) and SnoopTag (K742) and the catalytic residue in SnoopCatcher/SnoopLigase (E803) are indicated as green sticks.

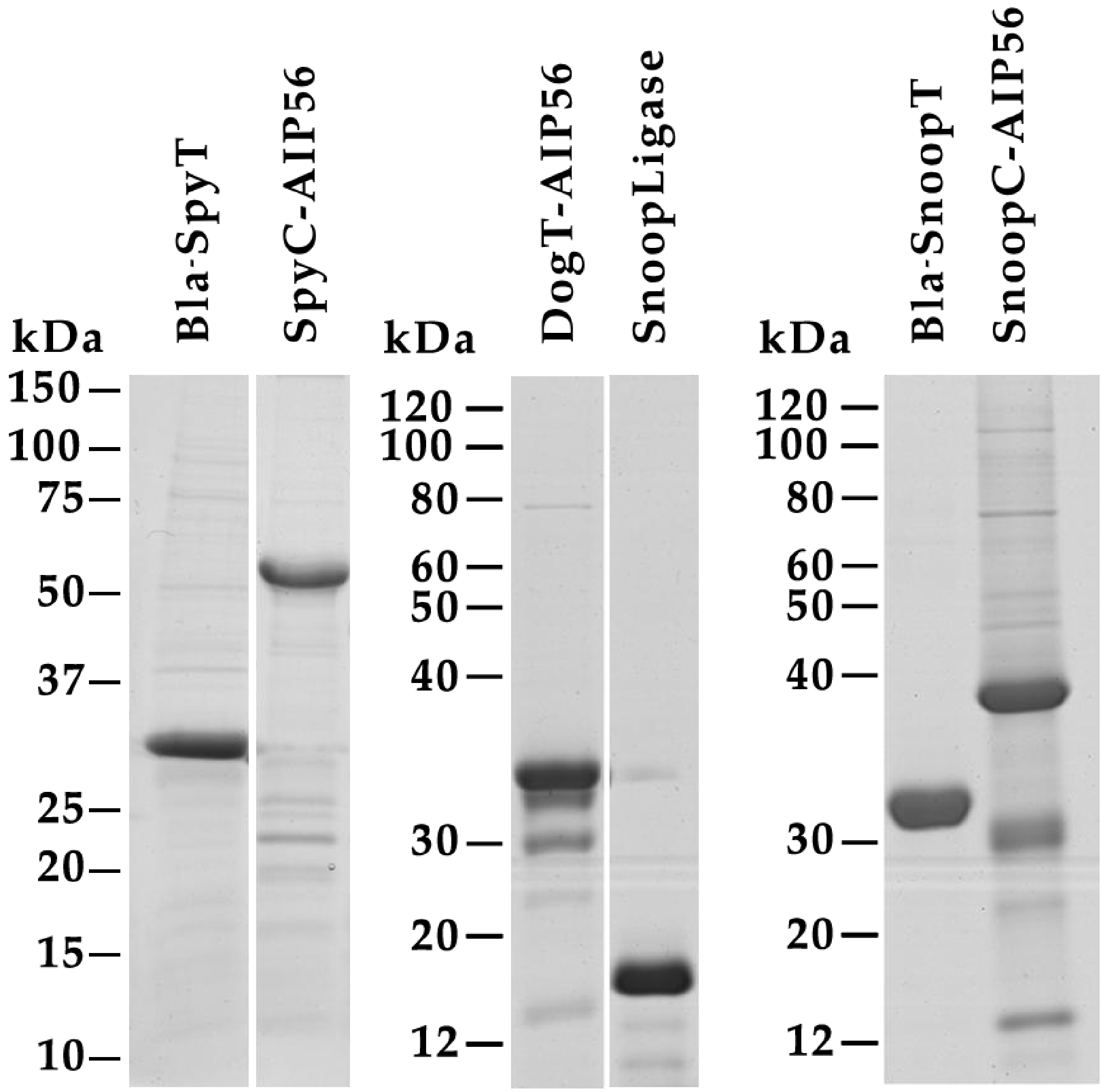

Supplementary Figure 2. SDS-PAGE profile of the purified recombinant proteins used for the fusion reactions. 5 (Bla-SpyT and SpyC-AIP56) and 10 (DogT-AIP56, SnoopLigase, Bla-SnoopT, SnoopC-AIP56) µg of protein were loaded and the gel stained with Coomassie-blue R-250. The positions of molecular weight markers are shown.

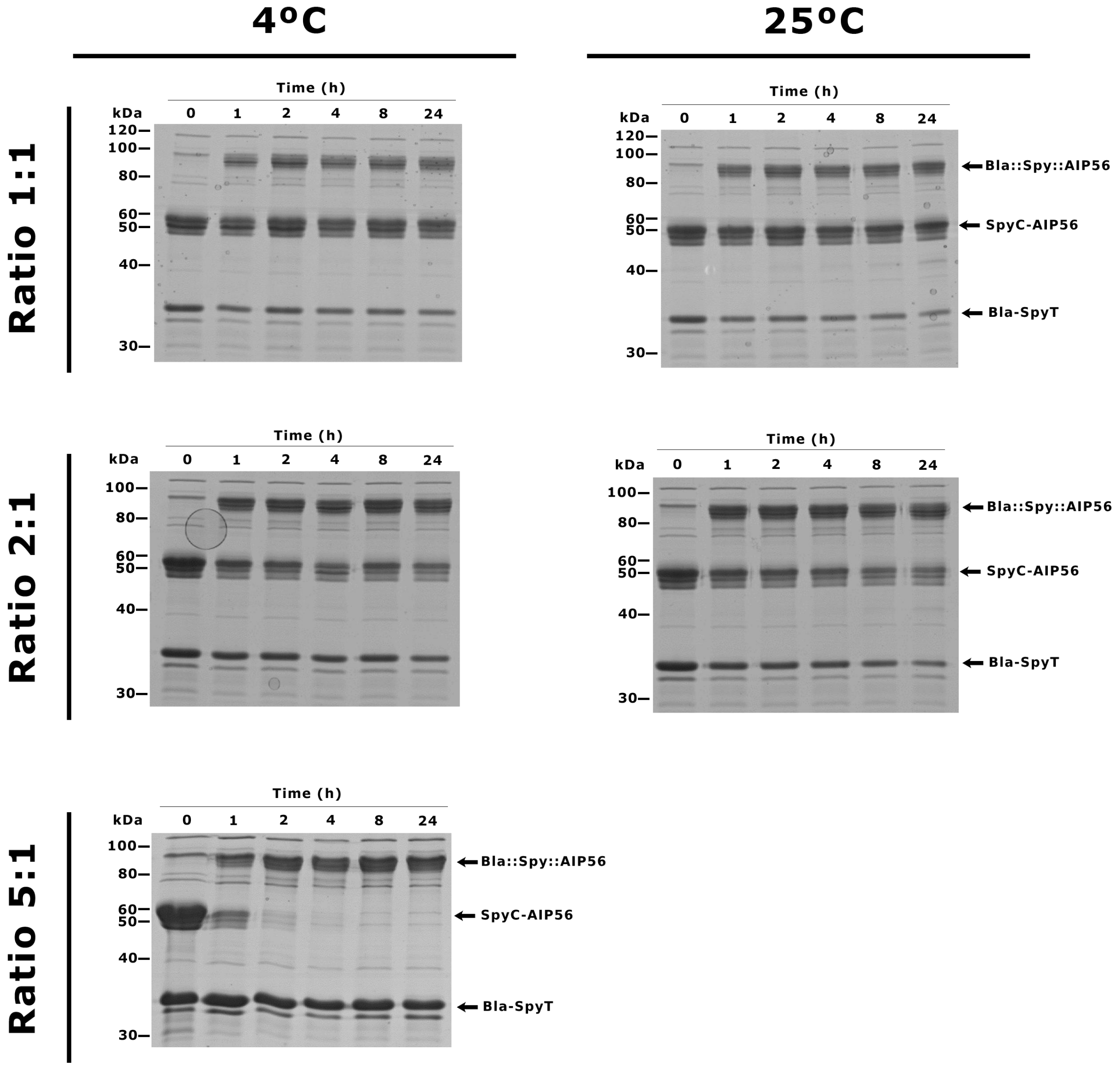

Supplementary Figure 3. Time-course analyses of the ligation of Bla-SpyT with SpyC-AIP56. Ligation reactions were performed at 4 °C or 25 °C and followed during 24 h, using different molar ratios of Bla-SpyT and SpyC-AIP56, as indicated. The gel for the best condition: molar ratio 5:1 25 °C (50 µM : 10 µM) - and the quantifications of chimera formation are presented in Figure 2a. Bla-SpyT (32.6 kDa) reacted with SpyC-AIP56 (41.3 kDa) to originate the Bla::Spy::AIP56 chimera of 73.9 kDa. Samples were subjected to SDS-PAGE followed by Coomassie-blue R-250 staining. Molecular weight markers are also shown.

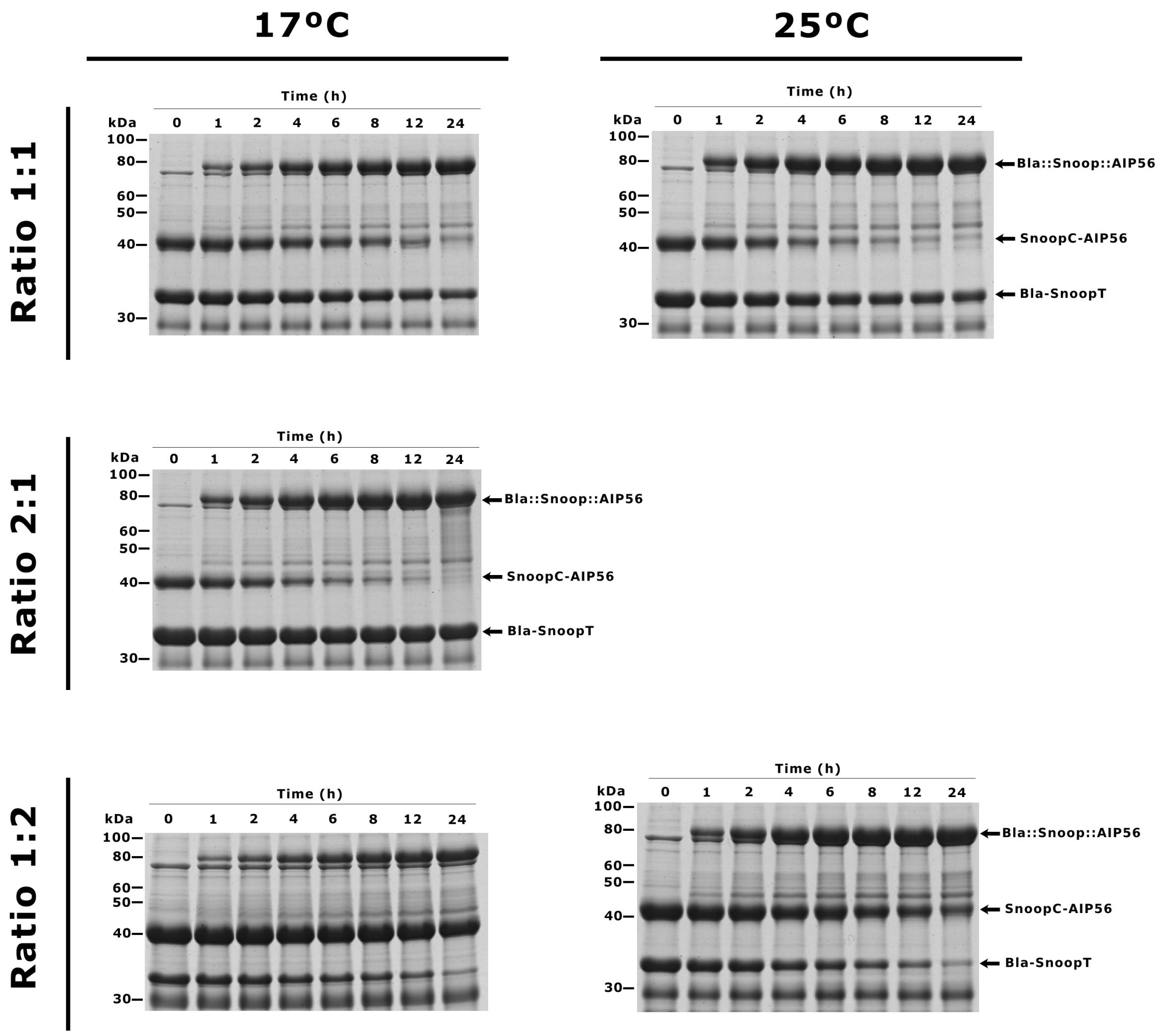

Supplementary Figure 4. Time-course analyses of the ligation reaction between Bla-SnoopT and SnoopC-AIP56. The two proteins were incubated at different protein ratios and temperatures, as indicated and followed during 24 h. The results obtained with a 2:1 ratio (20 µM : 10 µM) at 25 °C are presented in Figure 2b. Bla-SnoopT (31.8 kDa) reacted with SnoopC-AIP56 (41.2 kDa) to originate the Bla::Snoop::AIP56 chimera with an expected mass of 73.0 kDa. Samples were analyzed by SDS-PAGE followed by Coomassie-blue R-250 staining. Molecular weight markers are also shown.

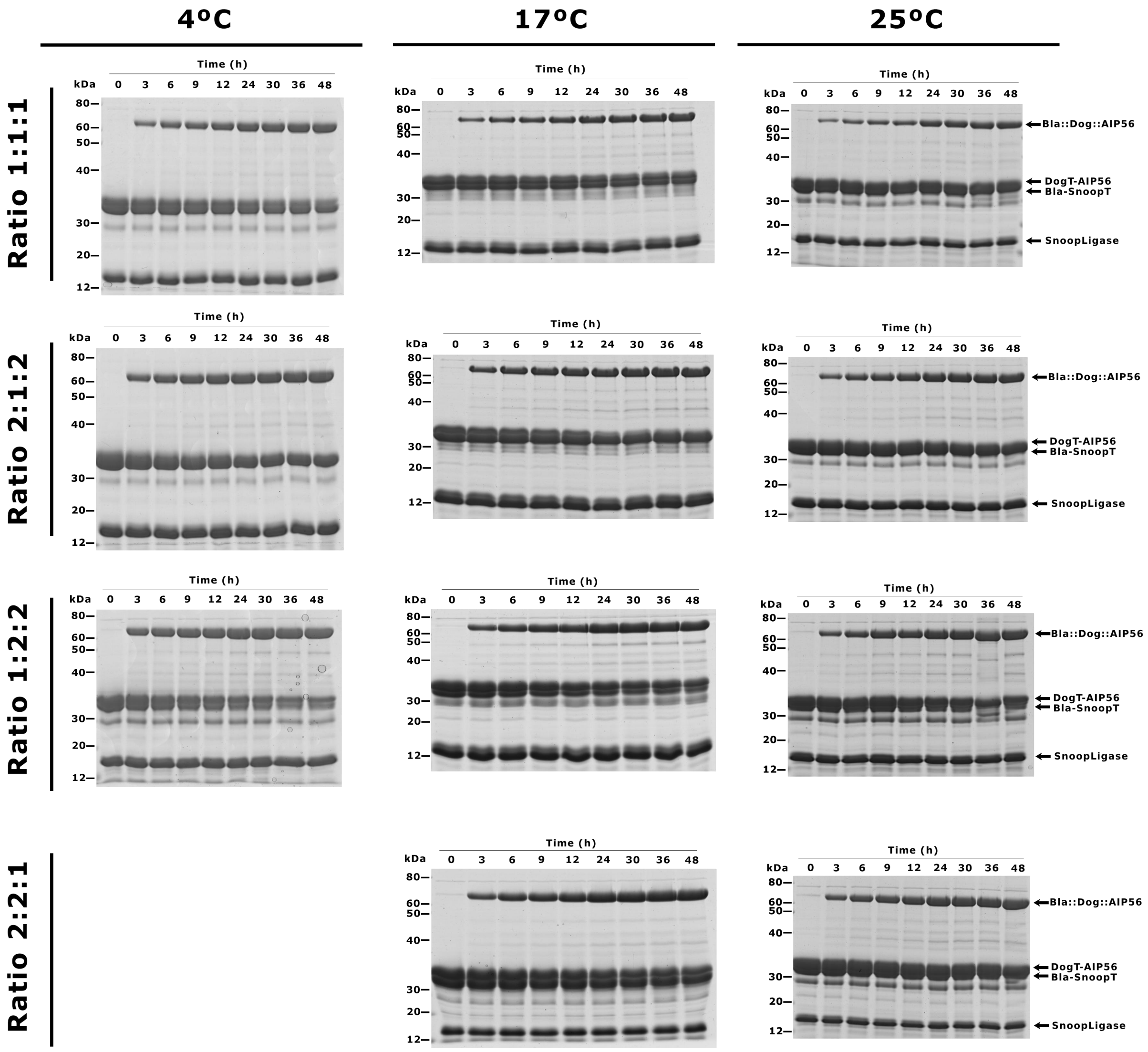

Supplementary Figure 5. Time-course analyses of the ligation reaction between Bla-SnoopT and DogT-AIP56. Bla-SnoopT and DogT-AIP56 were incubated at 4 °C, 17 °C or 25 °C at the indicated molar ratios and followed during 48 h. The results obtained at 4 °C with a 2:2:1 molar ratio (20 µM : 20 µM : 10 µM) are shown in Figure 2c. Bla-SnoopT (31.8 kDa) reacted with DogT-AIP56 (31.1 kDa) and SnoopLigase (15.4 kDa) to originate the Bla::Dog::AIP56 chimera with an expected mass of 63.0 kDa. Samples were analyzed by SDS-PAGE followed by Coomassie-blue R-250 staining. Molecular weight markers are also shown.

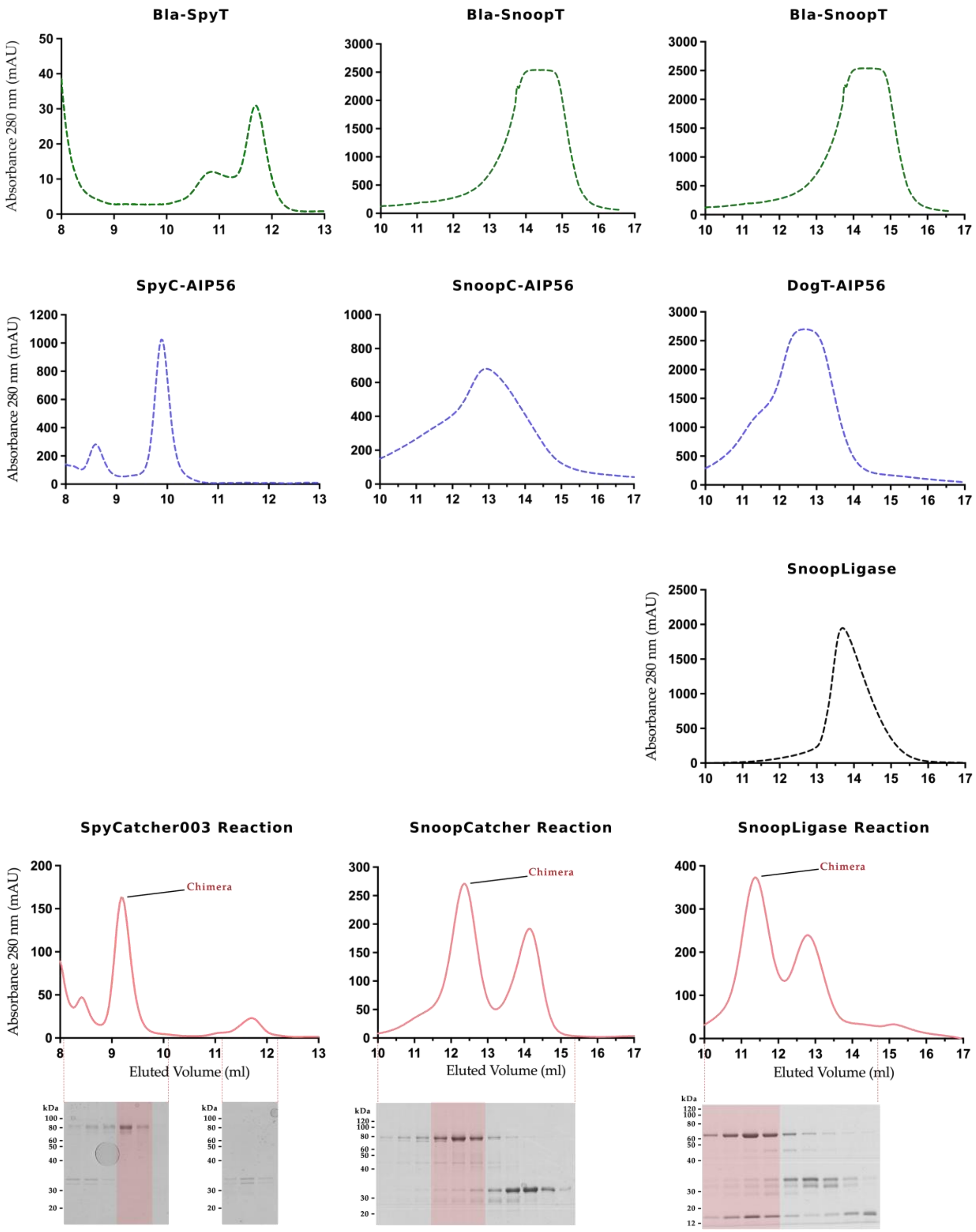

Supplementary Figure 6. Size-exclusion chromatography of the chimeras produced in the reactions. Chromatography was performed by injecting 500 µL of reaction into a Superdex 75 10/300 GL column (SpyCatcher003 Reaction) or Superose 12 10/300 GL column (SnoopCatcher/SnoopLigase Reaction). Columns were attached to an AKTA Purifier 10 FPLC system and the elution elapsed at 0.5 mL min/ml in 50 mM Tris pH 8.0, 300 mM NaCl. Chromatograms of all proteins used in the reactions are shown as reference. Fractions collected (highlighted in red) were analyzed by SDS-PAGE (Coomassie-staining).

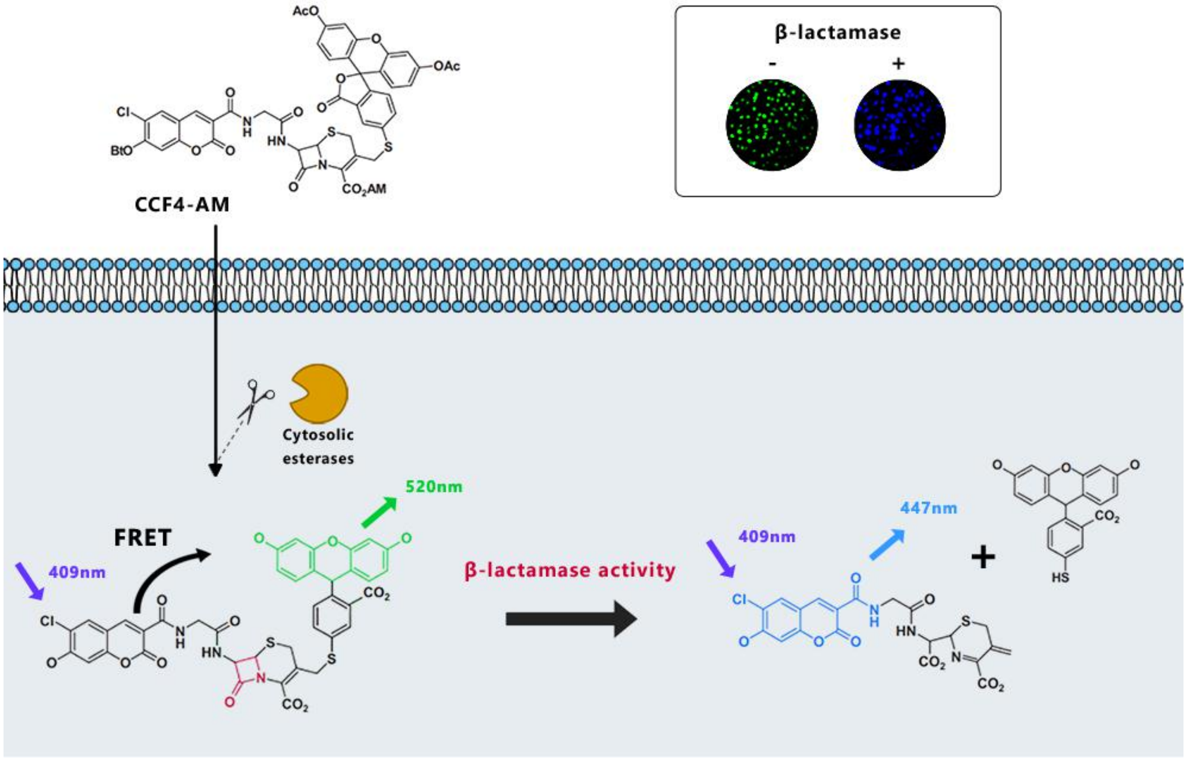

Supplementary Figure 7. β-lactamase FRET-based assay used in this work. The cell-permeable β-lactamase substrate CCF4-AM consists of a coumarin molecule connected to a fluorescein by a β–lactam ring. After incubation with the cells, CCF4-AM reaches the cytosol and its acetymoxymethyl tag (-AM) is cleaved by cytosolic esterases, leading to its retention in this compartment. In the absence of β–lactamase, excitation of coumarin at 409 nm results in FRET to fluorescein, and emission of green fluorescent light (520 nm). When β-lactamase reaches the cytosol, the β–lactam ring of CCF4 is cleaved, leading to FRET disruption and emission of blue fluorescent light (447 nm).

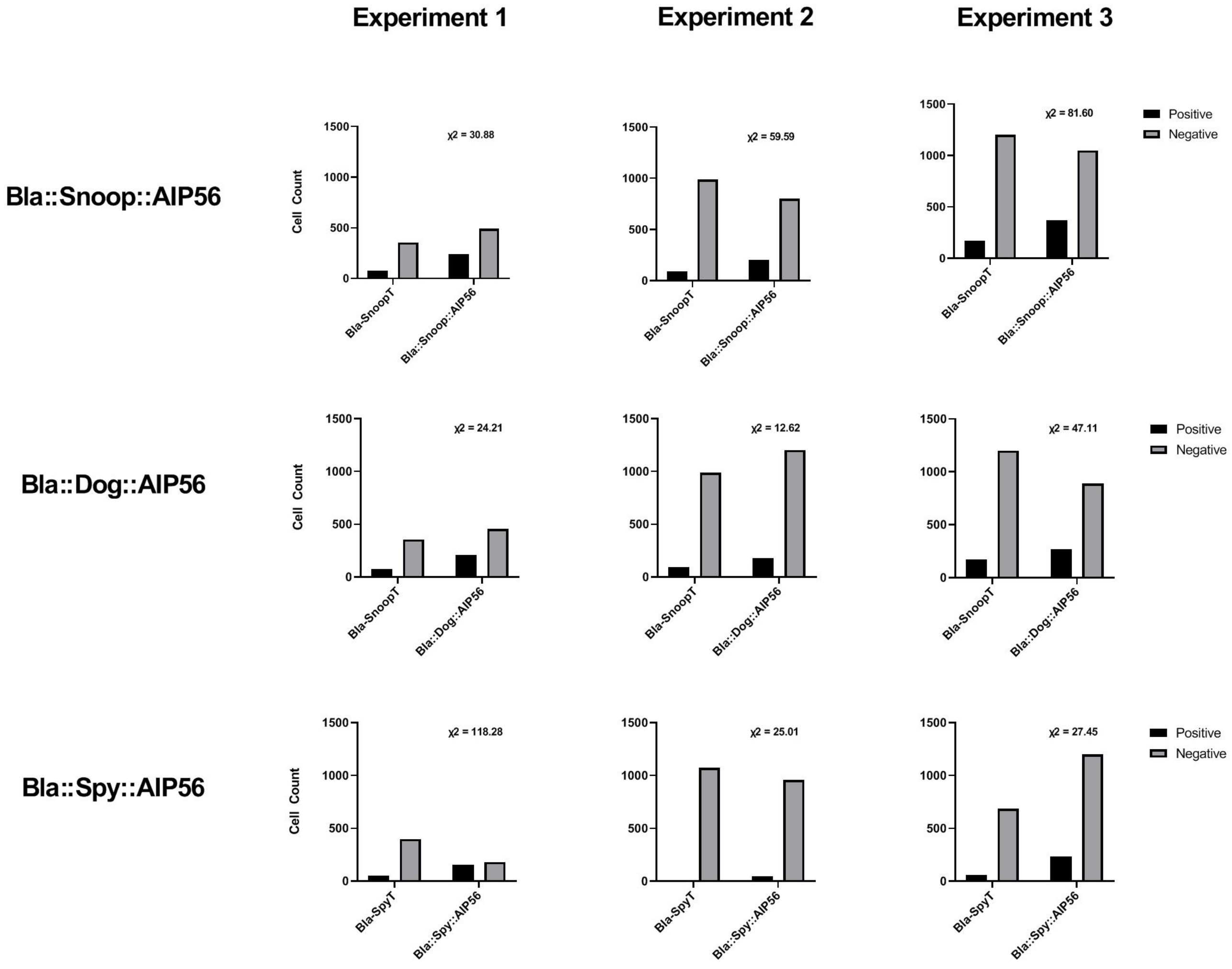

Supplementary Figure 8. Individual comparisons of cell positivity using chi-squared analysis. The number of positive and negative cells for each condition tested was compared using the chi-squared test with a confidence of 95 %. All individual comparisons were considered significant, with $p < 0.001$, in all experiments. Chi-squared critical value for df = 1 is 3.84.

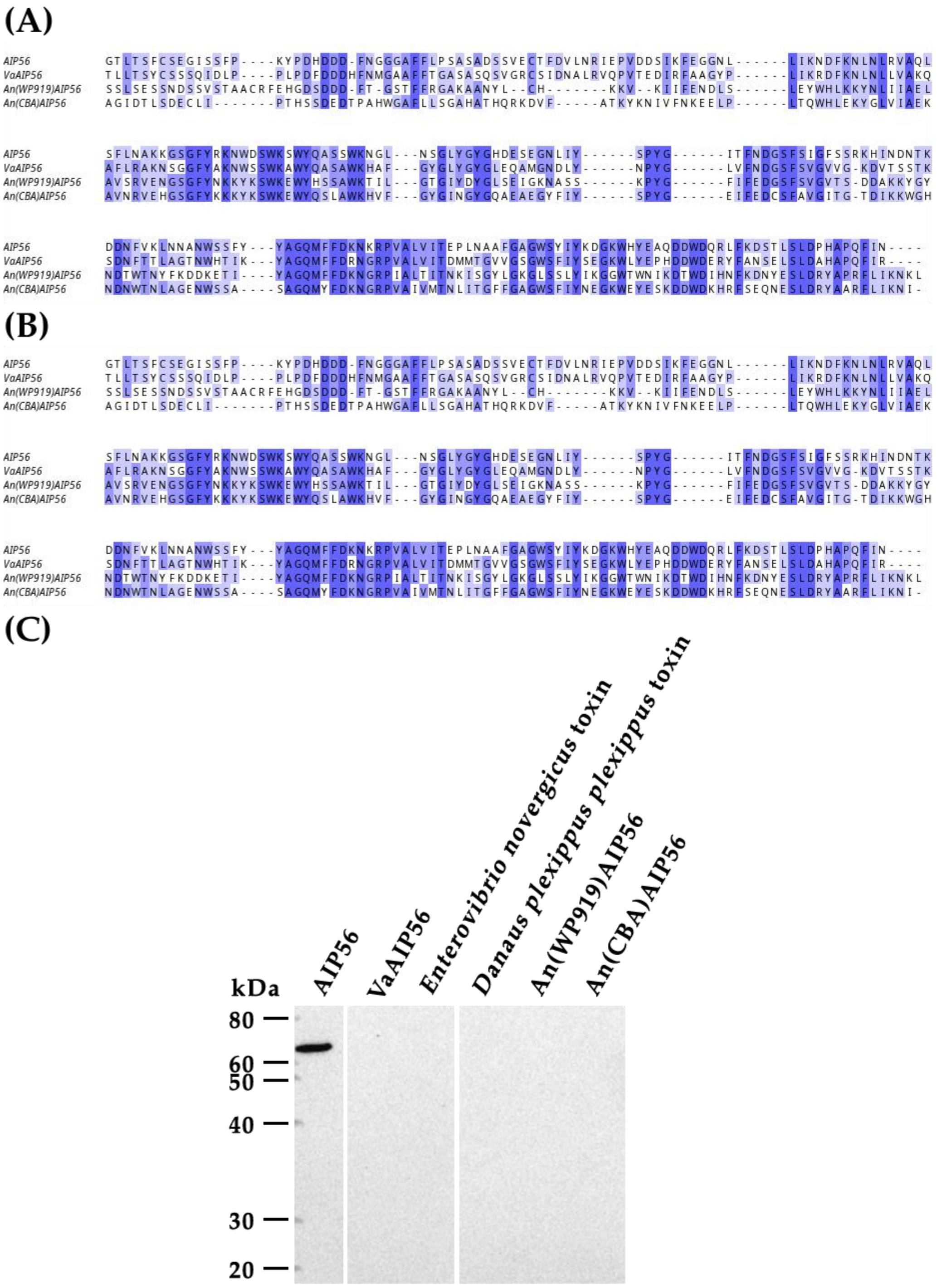

Supplementary Figure 9. Antibodies against AIP56 do not cross-react with AIP56-like and AIP56-related toxins. Alignment of **(A)** the delivery region of AIP56 with the C-terminal region of AIP56-like toxins from *Vibrio azureus* (VaAIP56, accession number WP_021710670) and *Arsenophonus nasoniae* [An(WP919)AIP56, accession number WP_051296919; and An(CBA)AIP56, accession number CBA76058], and **(B)** the delivery region of AIP56 with the C-terminal region of AIP56-related toxins from *Enterovibrio novergicus* (accession number WP_163391455) and *Danaus plexippus plexippus* (accession number

OWR44524). Alignments were performed using KAlign and residues with high identity were highlighted. Note the high amino-acid identity and similarity in both alignments. **(C) AIP56-like and AIP56-related toxins are not recognized by anti-AIP56 rabbit antibodies.** AIP56, VaAIP56, An(WP919)AIP56, An(CBA)AIP56, *Enterovibrio novergicus* toxin (middle and receptor-binding domains) and *Danaus plexippus plexippus* toxin (receptor-binding domain) were subjected to SDS-PAGE (100 ng/lane). Proteins were transferred to a nitrocellulose membrane that was then blocked for 30 min in blocking solution (5% (w/v) powder milk in Tris-buffered saline buffer + 0.1% (v/v) Tween 20 (TBS-T)) and incubated for 1 h at room temperature with a mixture of rabbit anti-$AIP56^{1-285}$ antibody (1:2500) and rabbit anti-$AIP56^{286-497}$ antibody (1:2500) in the same solution [30]. After washing with TBS-T, the membrane was incubated for 1 h with sheep anti-rabbit horseradish peroxidase (The Binding Site, AP311, 1:10.000 in blocking solution). Immunoreactive bands were visualized with a ChemiDoc Imaging System (BioRad) after incubation with Pierce ECL Western blotting substrate, according to the manufacturer's instructions. AIP56 was used as positive control. Expected molecular weights: AIP56 - 57.4 kDa, VaAIP56 - 61.3 kDa, *E. novergicus* toxin - 64.3 kDa, *D. plexippus plexippus* toxin - 21.1 kDa, An(WP919)AIP56 - 57.0 kDa, and An(CBA)AIP56 - 60.4 kDa.
